# Measurement of the hysteretic thermal properties of W-doped and undoped nanocrystalline powders of VO_2_

**DOI:** 10.1038/s41598-019-51162-4

**Published:** 2019-10-11

**Authors:** C. L. Gomez-Heredia, J. A. Ramirez-Rincon, D. Bhardwaj, P. Rajasekar, I. J. Tadeo, J. L. Cervantes-Lopez, J. Ordonez-Miranda, O. Ares, A. M. Umarji, J. Drevillon, K. Joulain, Y. Ezzahri, J. J. Alvarado-Gil

**Affiliations:** 1 Departamento de Física Aplicada, Cinvestav-Unidad Mérida, Carretera Antigua a Progreso km. 6, 97310 Mérida, Yucatán Mexico; 2 Materials Research Centre, Indian Institute of Science, 560012 Bengaluru, India; 3 Institut Pprime, CNRS, Université de Poitiers, ISAE-ENSMA, F-86962 Futuroscope Chasseneuil, France

**Keywords:** Mechanical engineering, Electronic devices

## Abstract

Hysteresis loops exhibited by the thermal properties of undoped and 0.8 at.% W-doped nanocrystalline powders of VO_2_ synthesized by means of the solution combustion method and compacted in pellets, are experimentally measured by photothermal radiometry. It is shown that: (i) the W doping reduces both the hysteresis loops of VO_2_ and its transition temperature up to 15 °C. (ii) The thermal diffusivity decreases (increases) until (after) the metallic domains become dominant in the VO_2_ insulating matrix, such that its variation across the metal-insulation transition is enhanced by 23.5% with W-0.8 at.% doping. By contrast, thermal conductivity (thermal effusivity) increases up to 45% (40%) as the metallic phase emerges in the VO_2_ structure due to the insulator-to-metal transition, and it enhances up to 11% (25%) in the insulator state when the local rutile phase is induced by the tungsten doping. (iii) The characteristic peak of the VO_2_ specific heat capacity is observed in both heating and cooling processes, such that the phase transition of the 0.8 at.% W-doped sample requires about 24% less thermal energy than the undoped one. (iv) The impact of the W doping on the four above-mentioned thermal properties of VO_2_ mainly shows up in its insulator phase, as a result of the distortion of the local lattice induced by the electrons of tungsten. W doping at 0.8 at.% thus enhances the VO_2_ capability to transport heat but diminishes its thermal switching efficiency.

## Introduction

Last decade thermochromic (TC) materials have been the focus of great interest because of their impressive ability to change reversibly electrical and optical properties with temperature. Among these, vanadium dioxide (VO_2_) represents one of the most interesting TC materials due to their fully reversible metal-to-insulator (MIT) transition at around 68 °C, which is a direct consequence of their structural transformation between low-temperature monoclinic to high-temperature tetragonal rutile phase^1^. At the MIT transition, abrupt changes in electrical resistivity^2–4^, electrical conductivity^5^, thermal properties^6–12^, optical transmittance and infrared emissivity^13–18^ of VO_2_ materials have been reported. Based on these characteristics, diverse technological applications have been proposed, such as temperature-sensing devices, optical- and electrical switching widgets and energy-efficient smart windows^19–21^. To enlarge its application range and to upgrade its properties, the reduction of the phase transition temperature (*T*_c_) of VO_2_ close to room temperature is highly desirable. Doping studies have revealed that the transition temperature can be tuned down by the incorporation of metal ions into the VO_2_ lattice, like tungsten (W) being one of the most effective donor level dopants^22–24^. In bulk and thin films, a decrease in *T*_c_ has been reported by an average of 20–26 °C per 1% in atomic weight (at) of W incorporated in the VO_2_ structure^25–29^. This is mainly associated with changes in: *i*. carrier density, since for charge compensation two W 3d electrons are transferred to the nearest neighbor V ion to form W^6+^–V^3+^ and V^4+^–V^3+^ pairs^22,28–34^, *ii*. lattice structure, generated by the large ion size of W respect to V, which promotes the formation of local rutile structure around W^6+^^22,30,31^, and *iii*. morphology and microstructure observed as a reduction in the grain size and shape^32^. Similarly, a decrease in the transition temperature of around 16–22 °C/at.% W for VO_2_ nanopowders tungsten-doped was found^32–35^, also accompanied by a considerably reduction in *T*_c_ at W = 0 at.% (~57 °C) thanks to the capability of the powdered material to get adapted to the temperature-induced-changes, due to the presence of void spaces among neighboring grains, which lessen the stress due to the phase changes^32,36^. In addition, in contrast to thin solid VO_2_ thin films production by sophisticated techniques, the preparation of powdered VO_2_ materials involve simple and low-cost methods which is suitable to be scaled up. These materials can be used for coating large surface substrates and with complex morphology at a wide range of thicknesses, from nanometers to micrometers, which would be adequate in industrial applications.

Inspired by the promising applications, several works have been developed to optimize the synthesis of VO_2_ nanopowders W-doped and undoped, and to perform their physical properties characterization through the metal-insulator transition^32–45^. It has been found that across the MIT, pure VO_2_ nanopowders prepared by thermolysis and hydrothermal methods exhibit excellent changes in electrical properties with a decrease (increase) in the resistance (conductivity) as large as two (three) orders of magnitude, in a reversible phase transition^34,41^. When doped with tungsten, VO_2_ nanopowders in their monoclinic phase show a reduction (growth) on the resistance (conductivity) being more relevant as W dopant concentration rises. However, in the tetragonal phase, both properties have the same order of magnitude of those found for undoped samples, which suggests that electrical properties of VO_2_ in its metallic state are little affected by the tungsten content. Significant reduction of the *T*_c_ and narrower hysteresis width (Δ*H*) were observed with the increasing of W dopant^34^. On the other hand, in the middle- and far-infrared spectral range, the thermochromic transition in optical transmittance of the VO_2_ nanocrystals significant falls when tungsten doping increases and when the crystals’ temperature rise, leading mostly hysteresis loops with narrow Δ*H* and lower transition temperatures^32,33,35,36,39,42–44^. Furthermore, in this optical approach, it has been found that infrared emissivity of VO_2_ nanopowders falls in about 33% across their phase transition, remaining constant in the extreme states^14,15^. As a function of the W content, Mao *et al*. studied the emissivity of VO_2_ powder films on cotton fabric along the MIT. For undoped samples a variation of 20% in the emissivity along MIT was found, declining respectively to 19%, 13% and 9% when W doping grows at 0.5, 1.0 and 1.5 at.%, which is also accompanied by a decrease in their critical temperatures (67, 57, 43 and 31 °C) and hysteresis widths (10, 9, 8 and 5 °C)^43^. In the case of the thermal properties, the transition enthalpy and the density of heat flow, which clearly reflect the phase transition performance, are strongly affected by the increase of W content in VO_2_ structure^32,35–38^. During heating (cooling) cycle, a big reduction in the enthalpy of 45% (44%) was found when comparing undoped and 1.8 at.% W-doped samples, which suggests that the W-doping content encourages the phase transition of VO_2_ powders^32^. Despite the heat flow in VO_2_ nanopowders has been already reported, the behavior of heat capacity has not been fully explored, which is certainly an analysis which would help in the design of performance of high-speed electronics. Likewise, thermophysical properties of VO_2_ powders, such as thermal conductivity and thermal diffusivity, have received much less attention, even though these properties drive the heat conduction in VO_2_ and therefore they may have a significant impact on energy conversion technologies. In particular, this is the case in biological applications based on novel photothermal converters used in pharmaceutical industry^46–48^. Given the importance of understanding the thermal behavior of VO_2_ and the notable advantages of W-doped VO_2_ nanopowders for the development of novel technologies, it is desirable to measure their thermal properties across the MIT and certainly, their characteristic thermal hysteresis.

In the present study, we measure the hysteresis loops of the thermal properties of VO_2_ nanocrystalline pressed powders W-doped around the MIT. This is done by photothermal radiometry (PTR), for VO_2_ powder samples, for 0 and 0.8 at.% tungsten content, synthesized by means of the solution combustion method and compacted in pellets using a hot-pressing treatment. We show that the MIT characteristics in the hysteresis loop of thermal properties, such as critical temperature and hysteresis width, can be reduced by doping VO_2_ lattice with tungsten. Moreover, we find that the thermal diffusivity of the pure VO_2_ pellet is slightly affected by the phase transition, but it considerably changes with the tungsten doping. In contrast, a remarkable increase in the thermal conductivity and thermal effusivity of both samples between their extreme metal-insulator states is found, while the W concentration only reduced slightly such changes. W doping thus leads to poor heat conduction switching efficiency in the VO_2_ transition, but interestingly this doping enhances the heat propagation in the insulating state and modifies the path that the thermal properties follow during the MIT. This behavior makes vanadium dioxide doped with tungsten a material with a greater flexibility than the undoped one, and this opens ways for novel applications.

## Methods and Materials

### Sample fabrication

#### V_2_O_5_ powder synthesis

(V_1−x_W_x_)_2_O_5_ (x = 0; 0.008) powder was synthesized by using the solution combustion method (SC), which was previously applied to fabricate V_1−x_W_x_O_2_ (0 ≤ x ≤ 0.02) thin films with a detailed structural, compositional and electrical characterization^29,49^. This efficient method is relatively less time-consuming than other ones to obtain oxide nanoparticles. An aqueous combustion mixture was made by dissolving ammonium metavanadate (NH_4_VO_3_, oxidizer), ammonium metatungstate (H_26_N_6_O_40_W_12_, oxidizer & source of W dopant) and Urea (CH_4_N_2_O, fuel) in dil. Nitric acid and water, respectively. The oxidizer to fuel ratio was maintained as 1. This aqueous combustion mixture was kept in a furnace preheated to 500 °C to initiate the combustion with rapid heating rate. The yellow powder obtained from self-propagating combustion reaction was ground using mortar and pestle. The obtained (V_1−x_W_x_)_2_O_5_ (x = 0, 0.008) powder was heat treated at 500 °C for 1 h to remove the residual carbon impurities.

### Hot pressing and reduction

(V_1−x_W_x_)_2_O_5_ (x = 0; 0.008) powder obtained from solution combustion was compacted as dense pellets using inductively heated home built hot press in argon atmosphere. Hot pressing was carried out at 580 °C for 6 minutes with 40 MPa pressure. Then, hot pressed pellets were subjected to reduction at 550 °C for 2 hours 30 minutes in N_2_ saturated with hydrocarbon atmosphere to get phase pure V_1−x_W_x_O_2_ (x = 0; 0.008) with nominal tungsten composition V_0.992_W_0.008_O_2_, that corresponds to 0.8 at.%. According to literature, W has solubility up to ~2 at.% in VO_2_^29^. W concentration in the nominal composition of our samples are well below the solubility (~2 at.%) limit. More details about sample fabrication can be found in ref.^49^. A summarize of the main characteristics of the VO_2_ pellets are shown in Table 1.

**Table 1:** Main characteristics about VO_2_ nanocrystalline powders.

	Undoped VO_2_	W-doped VO_2_
Diameter (mm)	12.51 ± 0.02	12.04 ± 0.02
Thickness (µm)	763.8 ± 6.7	672.2 ± 5.8
Compacted pressure (MPa)	40	40
Nominal tungsten (W) concentration (at.%)	0	0.8

### Sample structural characterization

**Figure 1:**
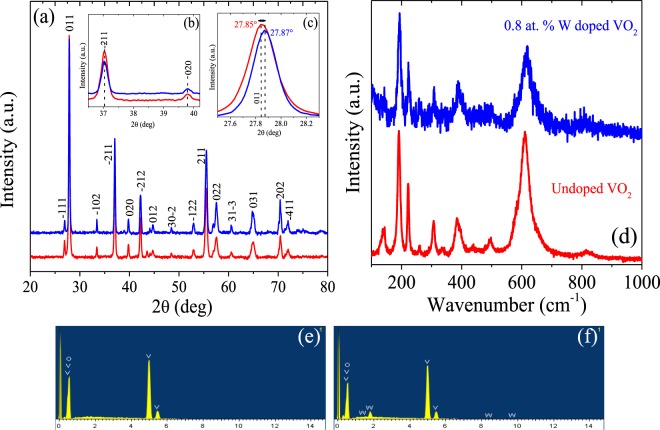
Room temperature XRD patterns of VO_2_ samples, with inset in the (**b**) (211), (020) and (**c**) (011) diffraction peaks. (**d**) Raman spectra of VO_2_ samples at room temperature. In red line the undoped pellet and in blue line the W-doped. (**e**,**f**) EDS patterns for undoped and W-doped VO2 samples, respectively.

Phase analysis of our samples has been carried out using X-Ray Diffraction (XRD) through a diffractometer Panalytical X’Pert PRO working with a Bragg-Brentano geometry and CuKα (λ = 1.5418 Å) radiation with 2*θ* range from 10° to 80° and the step size of 0.02°. XRD patterns of the undoped (red line) and W-doped (blue line) pellets are displayed in Fig. 1(a). The peaks detected for both samples can be attributed to a single monoclinic phase of VO_2_ according to the ICDD card. No. 04-003-2035. All peaks were intense and narrow, suggesting that samples were well crystallized. Some preferred orientation in the (011) direction was observed for the monoclinic VO_2_ samples. No characteristic peaks of tungsten oxides are observed, indicating that the W atoms enter the crystal lattice of VO_2_ rather than being in a separated crystalline phase. Furthermore, the characteristic slight shift in the (011) diffraction peak, previously reported in the literature for VO_2_ samples doped with W^32,37^, is also observed in Fig. 1(c) for our W-doped sample, which supports the presence of W in the VO_2_ pellet. No other diffraction peaks in the XRD pattern exhibit the shift (see Fig. 1(b)), as expected.

Additionally, Raman spectrum of the samples has been recorded between 50–1100 cm^−1^ using Horiba JobinYvon HR-Raman-123 micro PL spectrometer with a wavelength of 514 nm laser. Figure 1(d) illustrates Raman spectra of pure VO_2_ and VO_2_-W-doped pellets. Both Raman spectra show eleven bands located at 140, 191, 222, 261, 306, 339, 387, 441, 497, 614 and 822 cm^−1^ for undoped VO_2_ sample, which confirms the presence of VO_2_ with monoclinic M1 phase, and 143, 193, 224, 262, 309, 340, 389, 443, 499, 617 and 824 cm^−1^ for W-doped sample. This shift of wavenumber values towards high values with the incorporation of W in the VO_2_ matrix is consistent with previous reports^49^. Nevertheless, from the Raman spectrum of W-doped VO_2_ pellet, it can be seen that the peaks were significant less sharp and less strong with wider peak widths than those obtained for undoped VO_2_ sample. These results confirm that a local rutile structure is induced by the substitution of W atoms in the VO_2_ lattice, and then, the originally semiconductor phase of VO_2_ presents a partially metallic behavior.

The energy dispersive X-ray spectroscopy (EDS) analysis has been developed by means of JEOL JSM-7600F FESEM. EDS patterns of the undoped and W-doped VO_2_ samples are shown in Fig. 1(e,f), respectively. These results further confirm the existence of the V, W, and O elements. The representative peaks of the V and O elements appear in both samples, while the representative peaks of the W element only appear in W-doped product, which confirms a successful doping of W into VO_2_.

**Figure 2:**
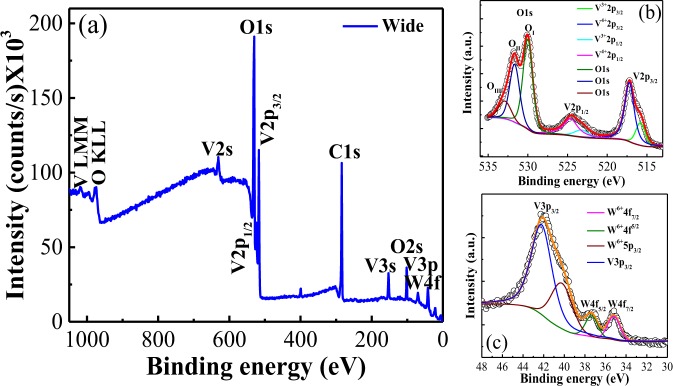
(**a**) XPS survey spectrum of the W-doped sample with high-resolution profiles for (**b**) V 2p, O 1s and (**c**) W 4 f. In (**b**) OI stands for the lattice oxide oxygen, OII and OIII are oxygens arising from surface contamination by atmospheric CO_2_ and H_2_O, respectively^60^.

Chemical composition and oxidation states of the samples were studied by X-ray photoelectron spectroscopy (XPS) (Fig. 2) by using a Thermo Scientific K-Alpha facility spectrometer. The survey XPS spectrum shown in Fig. 2(a), reveals the presence of three elements: V, W and O. The XPS analysis was conducted considering three different zones of our W-doped VO_2_ pellet. Furthermore, high-resolution profiles of the individual elements have been collected to quantify their amount in the sample (Fig. 2b,c). Quantification of tungsten is carried out considering the area under the peak and relative sensitivity factors (photoionization cross-section PIC) of the elements by using Eq. (1 S) and Table 1S presented in the supplementary material. Tungsten concentration in the sample is calculated to be 0.4 at.%. This concentration might be observed because of low penetration of X-rays making XPS as a surface sensitive technique and from the assumptions taken during the fitting process.

**Figure 3:**
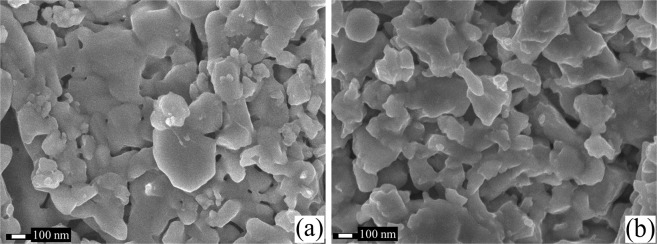
SEM micrograph of (**a**) undoped VO_2_ pellet and (**b**) W-0.8 at.% VO_2_ pellet.

The morphology of the VO_2_ pellets’ surface was studied by a Field Emission Scanning Electron Microscope (FESEM-JEOL-7600F). The SEM images obtained at 50k X are shown in Fig. 3(a,b) for undoped and W-doped samples, respectively. By comparing both images, a significant reduction in the crystalline grain size of around 150 nm is observed for the W-doped with respect to the undoped sample, which is consistent with previous results reported in the literature^32,35^. For both samples, crystals are seen as flattened structures as a consequence of the compacting force used to make the pellets. However, W doped pellet exhibits grains with more elongated shape and with a relatively lower connectivity than those observed in the undoped one.

**Figure 4:**
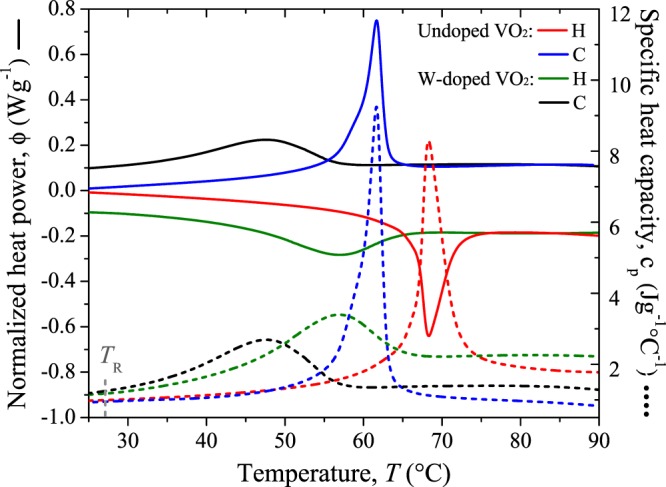
DSC profiles (continues lines) and specific heat capacity (dashed lines) of undoped VO_2_ and W-doped VO_2_ for the heating (red-green) and cooling (blue-black) cycles.

The typical DSC (Differential Scanning Calorimetry) curves of the VO_2_ nanopowders was performed by Discovery DSC- TA thermal analyzer under nitrogen flow in the range of 20 °C to 100 °C with a heating rate of 5 °C min^−1^. Thermal DSC profiles (normalized heat power) of our samples during the heating (red) and cooling (blue) procedures are shown Fig. 4 (continuous lines). Noticeable endothermal and exothermal peaks in the DSC curves, as a result of the phase transition, are observed for both samples. The transition temperature was found to be around 65.8 °C and 56.8 °C for the pure VO_2_ sample and W-doped one, respectively, which represents a reduction of 9 °C at 0.8 at.% of tungsten-doping. These results agree with those obtained in refs.^32,36,43^. Additionally, this DSC equipment provides the specific heat capacity that is presented in dashed lines in Fig. 4 for our VO_2_ pellets during their heating and cooling cycles. A noticeable reduction in the specific heat capacity of around 4.8 Jg^−1^ °C^−1^ (in heating) and 6.6 Jg^−1^ °C^−1^ (in cooling) for the W-doped VO_2_ sample is observed when comparing with the undoped one.

### Experimental setup

**Figure 5:**
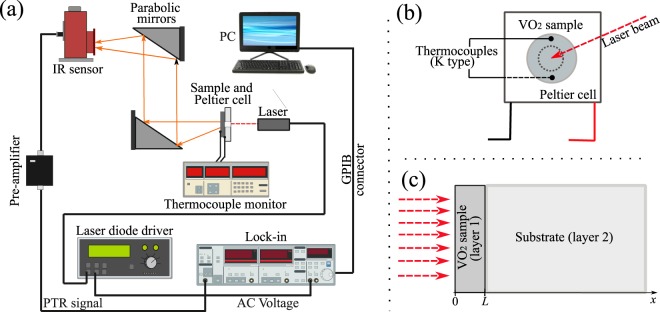
(**a**). General scheme of the experimental PTR system in the configuration of thermal-wave transmission, (**b**) scheme of the heating system and (**c**) schematic illustration of the layered system undergoing heat transport.

Photothermal radiometry (PTR) measurements performed at variable temperature were used to determine the thermal diffusivity and thermal effusivity of our VO_2_ pellets. A general scheme of the experimental system is presented in Fig. 5(a,b). In the transmission (reflection) experimental configuration of the PTR technique, the rear (front) surface is illuminated with a modulated laser beam at a frequency *f*, resulting in periodic temperature fluctuations of the sample, which in turn causes an emission of infrared light train collected by means of two parabolic mirrors and then sent into an IR sensor. The photothermal signals detected depend mainly on the thermal diffusivity, thermal effusivity and emissivity of the sample, for which this technique has been used extensively to develop thermal^50,51^ and optical^52–54^ characterizations of a large variety of materials. Especially, the use a lock-in amplifier and filters make this photothermal technique very reliable and accurate in measuring very low signals even in highly noisy environments. In our experiment, the VO_2_ pellet (12 mm in diameter) is illuminated on the back surface (transmission experimental mode) by a diode laser beam (250 mW, λ = 808 nm), with a spot diameter *D = *5 mm, working at a fixed modulation frequency of 5 Hz. The infrared radiation emitted by the frontal surface is collected and focused by two Edmund optics EFL 90° protected aluminum parabolic mirrors and sent towards the IR sensor Judson J15D12 HgCdTe (detection range 2 to 12 µm) that detects the changes in the power radiation emitted by the sample ($\Delta W$). The measured voltage signal is preamplifed (Vigo PPS-02) and sent into a lock-in amplifier (Stanford Research Systems SR-830 DSP) to be finally stored as amplitude and phase in a computer. The temperature of the VO_2_ sample (*T*) during its heating and cooling is respectively controlled and measured with a Peltier cell (*V*_max_ = 5.3 V, *I*_max_ = 5.7 A, Δ*T*_max_ = 68 °C) and K-thermocouples (0.05 mm in diameter) placed on both faces of the sample as shown in Fig. 5(b), which in turn are connected to a thermocouple monitor (Stanford Research Systems SR-630) with 0.1 °C of resolution. The reported amplitude and phase signals correspond to an average of five repetitions at each fixed temperature, and the sample temperature is an average of the temperature measured on each sample’s face.

### Theoretical model

Heat propagation through the layers shown in Fig. 5(c) is expected to satisfy the one-dimensional condition^53^ because of the parameters used in this work (laser spot *D* and modulation frequency *f*). By assuming the layered system as an opaque single layer (layer 1) in thermal contact with a semi-infinite medium (layer 2), the harmonically modulated front (*F, x* = 0) and rear (*R, x* = *L*) surface temperature is given by^55^:

1 $$\theta (x)=\frac{{I}_{0}}{{k}_{1}{\sigma }_{1}}[\frac{(1-{b}_{21}){e}^{{\sigma }_{1}(x-L)}+(1+{b}_{21}){e}^{-{\sigma }_{1}(x-L)}}{(1+{b}_{21}){e}^{{\sigma }_{1}L}-(1-{b}_{21}){e}^{-{\sigma }_{1}L}}]+S(f),$$

where ${I}_{0}$ is the intensity of the laser beam, $S(f)$ is the transfer function of the experimental setup, ${b}_{21}={\varepsilon }_{2}/{\varepsilon }_{1}$ is the ratio of thermal effusivities of the layers, $\varepsilon =k/\sqrt{\alpha }$ with *k* thermal conductivity and *α* thermal diffusivity, ${\sigma }_{1}=(1+i)/{\mu }_{1}$ and ${\mu }_{1}=\sqrt{\pi f/{\alpha }_{1}}$ are the thermal complex diffusion coefficient and the thermal diffusion length of the layer 1, that strongly attenuates the thermal-wave propagation. This latter parameter is experimentally controllable and allows developing thermal depth profiles in both the thermally thin (*L* ≪ *μ*) and thermally thick (*L* ≫ *μ*) regimes of diffusive heat transport.

On the other hand, it has been demonstrated that the change in the infrared radiation emitted ($\Delta W$) by a sample’s surface, that corresponds to the quantity directly measured by our sensor, can be written as:

2 $$\varDelta W=4{\epsilon }{\sigma }_{B}{T}^{3}\theta (x),$$

where *ε* being the emissivity of the sample, ${\sigma }_{B}$ is the Stefan-Boltzmann constant and *T* is the steady-state temperature rise of the surface. Through this dependence, the radiometric signal also depends on the sample emissivity.

### Methodology

**Figure 6:**
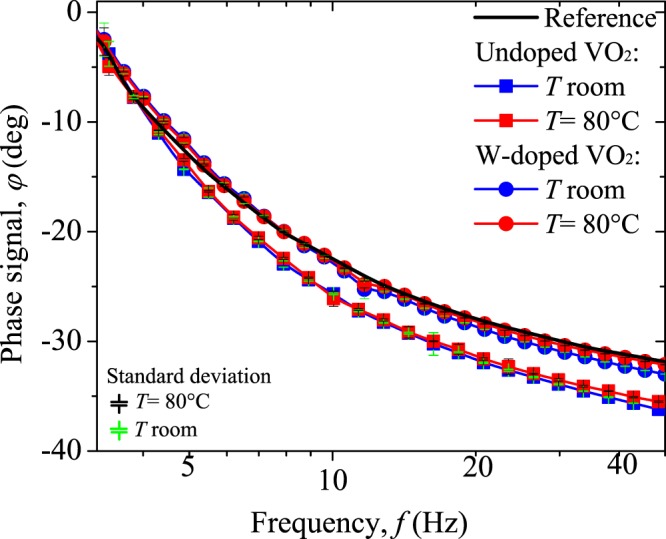
Phase signal obtained by the modulation frequency scanning method for undoped sample (squares) and W-doped one (circles) at room temperature (blue) and at 80 °C (red) in the reflection configuration. Black line represents the thermal profile of a thermally thick reference.

To establish the regime of diffusive heat transport in which the experiment will be developed, we measured in the reflection experimental configuration the radiometric signals as the frequency of the laser beam intensity is varied from 3 to 50 Hz, when the sample is at room temperature and at 80 °C. Phase signals of our VO_2_ pellets at both temperatures are presented in Fig. 6, as well as the thermal profile of a thermally thick sample used as a reference (Sigradur K- Glassy Carbon – HTW, thickness 2 mm). From Fig. 6, it can be seen that phase signals obtained for the VO_2_ pellets on both insulator and metallic states, exhibit similar profiles to the one of the reference, that corresponds to the transfer function associated with the electronics of our experimental setup^55^. This implies that this radiometric signal is quite independent of the thermal properties of the material, particularly at low frequencies, such as predicted by the model of Eq. (1) for a single layer in contact with a semi-infinite medium in the thermally thick regime^53,55,56^. Therefore, thermally thick regime (*L* >  > *μ*) and one-dimensional heat propagation are appropriate approximations for this layered system at 5 Hz, that is the value of the fixed modulation frequency in our experiments. Based on these arguments, we can reduce Eq. (1) for reflection (*F*) and transmission (*R*) modes as follows:

3a $${\theta }_{F}(x=0)=\frac{{I}_{0}}{{k}_{1}{\sigma }_{1}}+S(f)$$

3b $${\theta }_{R}(x=L)=\frac{{I}_{0}}{{k}_{1}{\sigma }_{1}}{e}^{-\sigma L}+S(f)$$

Analyzing the amplitude and phase of the temperature distribution functions displayed in Eq. (3), it is found that: *i*. the amplitude obtained is inversely proportional to the thermal effusivity of layer 1 for both reflection and transmission modes; though this latter also depends on the thermal diffusivity, and *ii*. the phase signal is independent of these parameters, remaining constant in the reflection configuration, while it has a strong dependence on the thermal diffusivity in the transmission one. Taking into account that transmission measurements can provide information about both thermal diffusivity $\alpha (T)$ and thermal effusivity $\varepsilon (T)$ of the sample, we have developed and analyzed the experiments mainly in that configuration. To eliminate the transfer function dependence $S(f)$, the amplitude *A* and phase $\phi$ signals obtained at each temperature *T* are normalized with their counterpart at room temperature *T*_0_, whereby normalized signals have a dependence with $\alpha ({T}_{0})$ and $\varepsilon ({T}_{0})$ as shown in Eq. (4).

4a $${A}_{{\rm{N}}}(T)=\frac{{T}^{3}}{{T}_{0}^{3}}\frac{{e}^{-L/\mu (T)}}{{e}^{-L/\mu ({T}_{0})}}\frac{\varepsilon ({T}_{0})}{\varepsilon (T)}$$

4b $$\tan \,{\phi }_{{\rm{N}}}(T)=\frac{1+\,\tanh (x)\cot (x)}{1-\,\tanh (x)\cot (x)}-\frac{1+\,\tanh ({x}_{0})\cot ({x}_{0})}{1-\,\tanh ({x}_{0})\cot ({x}_{0})}$$

where $$x=\frac{L}{\mu (T)},{x}_{0}=\frac{L}{\mu ({T}_{0})}\,\,$$ and $$\mu (T)=\sqrt{\frac{\alpha (T)}{\pi f}},\,\mu ({T}_{0})=\sqrt{\frac{\alpha ({T}_{0})}{\pi f}}.$$

The experimental values of the normalized amplitude (normalized phase) have been fitted with ${A}_{{\rm{N}}}$ ($\tan \,{\phi }_{{\rm{N}}}$) to determine the thermal effusivity (thermal diffusivity) of our VO_2_ pellets at different temperatures within their MITs. Thermal conductivity and specific heat capacity at constant pressure are obtained from the relations: $k=\varepsilon \sqrt{\alpha }$ and ${c}_{{\rm{p}}}=k{(\rho \alpha )}^{-1}$ respectively, where $\rho =$ 4571 kg m^−3^ is the standard VO_2_ density.

## Results and Discussion

**Figure 7:**
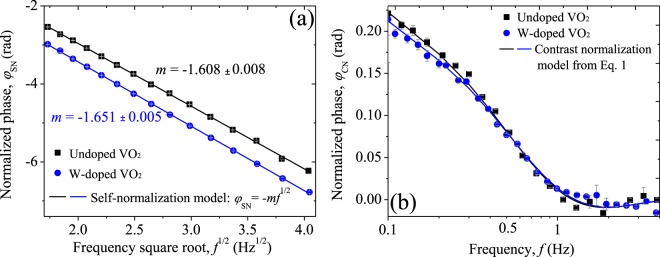
Normalized phase signal in a frequency-scan obtained by (**a**) self-normalization and (**b**) thermal contrast methodologies. In gray lines standard deviation.

In order to find the thermal diffusivity and thermal effusivity of our VO_2_ samples across their insulator-to-metal transition, it is necessary to know these properties at room temperature according with Eq. (4), which can be accurately and reliably found by means of the well-established self-normalization and thermal contrast methods, commonly used in PTR technique^56,57^. In the first method, the signal measured at the rear surface (transmission) is normalized with that obtained at the front face (reflection), leading in the thermally thick limit a linear dependency between the normalized phase ${\phi }_{{\rm{SN}}}$ and the square root of frequency ${f}^{1/2}$. Thermal diffusivity of the samples can be obtained from the slope/fitting parameter $m={(\pi {\alpha }^{-1})}^{1/2}L$, as is done in Fig. 7(a). On the other hand, in the second method, a thermal contrast between two-layer systems: layer/medium 1 and layer/medium 2 is carried out. Normalized phase ${\phi }_{{\rm{CN}}}$ has been calculated from the phase signal measured for the sample/air and sample/water systems in a frequency-scan as shown in Fig. 7(b). By fitting the experimental data (dots) with the appropriated model (line), derived from Eq. (1), the thermal effusivity of the samples at room temperature is found. It is worth mentioning that samples’ surfaces were coated with a layer of ~30 nm of AuPd to avoid the absorption of water. Additionally, in this experiment we consider room temperature as *T*_0_ = 27 °C due to the contribution of the laser’s heating on the sample temperature. Thermal properties obtained for each sample at *T*_0_ are summarized in Table 2.

**Table 2:** Thermal properties of undoped and W-doped VO_2_ samples at room temperature.

	Thermal diffusivity, α (mm^2^ s^−1^)	Thermal effusivity, *ε* (kWs^1/2^ m^−1^ K^−1^)	Thermal conductivity, *k* (Wm^−1^ K^−1^)	Specific heat capacity, *c*_p_ (J g^−1^ K^−1^)
Undoped VO_2_	0.707 ± 0.006	7.53 ± 0.15	6.33 ± 0.14	1.96 ± 0.04
W-doped VO_2_	0.530 ± 0.004	9.70 ± 0.29	7.06 ± 0.23	2.96 ± 0.09

From Table 2 we can affirm that at room temperature W doping improves the ability of the VO_2_ pellet to conduct heat and to transfer thermal energy with its surroundings, as a result of the increase in its thermal conductivity and thermal effusivity. However, the thermal energy diffusion rate related with the thermal diffusivity decreases, while the amount of heat per unit mass required to raise the temperature by 1 K, known as a specific heat capacity, grows. This latter result is supported by the DSC profiles presented in Fig. 4 (gray dotted line) evaluated at *T*_0_. In addition, note that for undoped VO_2_ sample the value of *k* agrees with those obtained in other works^6–8^.

**Figure 8:**
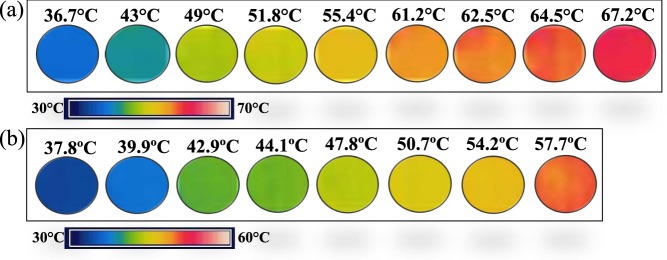
Thermal infrared images taken from FLIR camera of (**a**) undoped VO_2_ and (**b**) W-doped VO_2_ samples during the heating process.

On the other hand, it is well known that VO_2_ materials across the insulator-to-metal (metal-to-insulator) transition usually exhibit a decline (increment) in their emissivity *ε*^13–18^, which in our case could strongly affect the measured PTR signal as sample temperature is varied according with Eq. 2. However, in VO_2_ pellets, the change in the emissivity along the MIT highly depends on the force used to compact the VO_2_ powder as was demonstrated in refs.^14,15^. Thus, greater compacted forces induce more coupled grains and in consequence higher emissivity variations. In order to estimate the variation in our emissivity samples, thermal infrared images have been taken during the heating procedure by a FLIR camera A-20 (8–14 μm) as shown in Fig. 8. Essentially, infrared camera detects the radiation emitted by the sample surface, which depends on its temperature and emissivity. In our case, *ε* is fixed at 0.85 while *T* of the VO_2_ pellets is increased. Therefore, the changes in the radiation detected by the IR camera, which appear as a decrease in temperature, are the result of variations in the sample emissivity^17,25,40^. Based on this, the linearity in temperature observed in the thermal images taken for undoped (Fig. 8a) and W-doped (Fig. 8b) VO_2_ pellets, guarantees that the emissivity of our samples does not change throughout the MIT. This result was expected due to the relative very low pressing force used to compact our samples compared to other reported works^14,15^.

**Figure 9:**
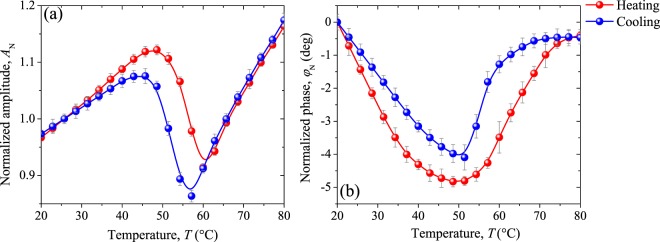
(**a**) Normalized amplitude and (**b**) normalized phase signals measured during the heating and cooling of undoped VO_2_ pellet. In gray lines the standard deviation.

In agreement with Eq. (4), thermal properties of our VO_2_ pellets during the MIT can be found measuring the PTR signals at a fixed frequency by scanning the sample temperature. The normalized amplitude (NA) and normalized phase (NP) measured for the undoped VO_2_ sample across the MIT are presented in Fig. 9(a,b), respectively. Note that NA increases linearly at temperatures outside the MIT in agreement with the temperature dependence predicted by Eq. 2, while NP seems to fall linearly before the transition, and after this, tends to remain constant. The independence of this behavior with the cycle (heating/cooling) for low and high temperatures in both signals, indicates that the sample has reached its dielectric and metallic state, respectively. However, the temperatures at which sample is switching into those states in amplitude signal differs from the phase signal. This is expected, since according to Eq. (4), the PTR signal depends on the different thermal properties. VO_2_ physical properties manifest different dependence with the temperature, leading to differences in their transition temperatures, in agreement with the results reported in the literature^17^.

Within the MIT, both signals vary meaningfully, taking different values for the heating and cooling processes, which shows that they are sensitive to the thermal hysteresis of our undoped VO_2_ sample. Critical temperature calculated by the peaks of the derivative amplitude and phase was found to be around 55.1 °C and 57.1 °C, respectively.

**Figure 10:**
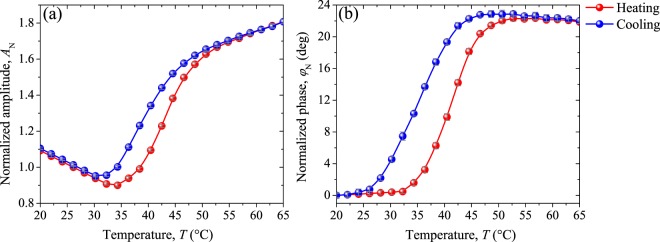
(**a**) Normalized amplitude and (**b**) normalized phase signals measured during the heating and cooling of W-doped VO_2_ pellet. In gray lines the standard deviation.

Figure 10 shows the evolution of the normalized amplitude and normalized phase recorded for the W-doped VO_2_ pellet around its *T*_c_, determined to be 39.5 °C from both signals. Thus, the well-known reduction in the critical temperature when VO_2_ lattice is doped with tungsten is observed. A variation of around 16.6 °C between 0 and 0.8 at.% W doping level is found, which is consistent with previous results reported^27,32,34^. Note also that the behavior of the signals throughout the MIT for VO_2_ W-doped (Fig. 10), is mostly similar to that found for undoped VO_2_ sample (Fig. 9) in the temperature range from the middle of the transition (~50 °C) to the metallic state (80 °C). It can be attributed to the reduction in the crystalline distortion induced by the W atoms in the monoclinic phase, which shifts the density of states in such a way available states in the conduction band grows, inducing a decrease in the activation energy required to change the phase^22,30,31^. That means that, substitutional tungsten doping promotes the metallic domains nucleation in the VO_2_ insulating matrix, facilitating the percolative insulator-metal transition.

Evaluating in detail the normalized radiometric signals presented in Figs 9 and 10 at the extreme stages of the metal-insulator transition, it can be observed that unlike to the behavior of the metallic state, where normalized amplitude (phase) increases linearly (remains constant) for both samples, in the insulating one, they drastically change. This occurs in such a way that, in undoped sample NA (NP) increases (decreases) while in W-doped one decreases (remain constant). In addition, note that in average, larger variations in the NA and NP signals during the MIT were found for W-doped sample (0.9, 21.7 deg) than for undoped one (0.2, 4.4 deg).

**Figure 11:**
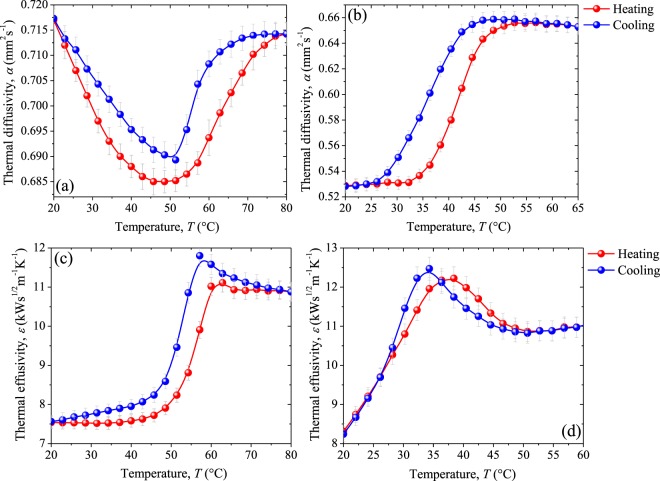
Thermal diffusivity and thermal effusivity of (**a**–**c**) undoped VO_2_ sample, and (**b**–**d**) W-doped VO_2_ sample as a function of their temperatures for the heating (red) and cooling (blue) procedures. In gray lines the absolute error.

Thermal diffusivity *α* of our undoped VO_2_ and W-doped VO_2_ pellets are respectively shown in Fig. 11(a,b). In the MIT, the variations in *α* during the heating and cooling cycles follows a different path, which leads to an hysteresis loop similar to its corresponding radiometric signals displayed in Figs 9 and 10. For undoped sample, thermal diffusivity falls (rises) until (after) 50 °C varying just 4.5% (4%) with respect to its value at room temperature. This particular behavior in thermal diffusivity agrees with the one that can be predicted from the thermal conductivity^6,7^ and specific heat capacity^6–8,11^ for VO_2_ materials across the MIT reported in previous works. Interestingly, as is shown in Fig. 11(a), in our samples of undoped VO_2_, thermal diffusivity is almost the same for the insulating (20 °C) and metallic states (80 °C), with a difference smaller than 1%.

A notable feature of the dependence of the thermal diffusivity for the doped and undoped samples can be noticed by observing, in Fig. 11(a), the evolution of the thermal diffusivity for the heating cycle. Above 48 °C, it remains nearly constant for a few degrees and after that the thermal diffusivity starts to grow rapidly up to reaching the metallic phase, where the thermal diffusion stabilizes. This tendency matches remarkably the trend of the thermal diffusivity during the entire transition of W-doped VO_2_ sample (Fig. 11b). This behavior supports the idea that W doping induces the creation of conductive regions in the crystalline structure of the VO_2_ pellet, which can be better appreciated in the dielectric state (under 48 °C), that now behaves as a partially-metallic phase. This is why the thermal diffusivity of W-doped pellet only increases (decreases) across the MIT in the heating (cooling) procedure, remaining almost constant in the pure states.

An important fact that can also be observed in Fig. 11(a,b), thermal diffusivity for the W-doped material presents a significant variation of around 24% in the MIT. Comparing the thermal diffusivity of doped and undoped samples, we can realize that the emergence of metallic domains in the VO_2_ insulating matrix due to the tungsten doping, substantially disrupts the capacity of efficiently transfer heat carriers through the material. Similarly, for the undoped sample, the thermal diffusivity before 45 °C, falls when the temperature increases; in these temperature range, metallic islands begin to rise in the semiconductor matrix making heat transfer inefficient.

It is interesting to note that doping promotes higher variations in *α* along the MIT with narrower hysteresis ($\Delta H$ = 6.1 °C) than those obtained for pure VO_2_ ($\Delta H$ = 9.5 °C), which can be useful to control and improve the switching operation of the heat propagation velocity in many fundamental devices.

Figure 11(c,d) show the thermal effusivity *ε*, measured along the phase transition, for undoped VO_2_ and W-doped VO_2_ samples respectively. As it happens for radiometric signals (Figs 9 and 10) and for thermal diffusivity (Fig. 11a,b), the trend of thermal effusivity with the temperature for undoped VO_2_ differs from the W-doped one in the dielectric phase. At temperatures lower than 43 °C (27 °C) for undoped (doped), *ε* tends to be constant (grow linearly). After that, in the MIT, thermal effusivity for undoped (doped) VO_2_ rises at around 44.3% (34.4%) until reaching its metallic state at *ε*_m etallic_ = 10.9 (10.8) kWs^1/2^m^−1^K^−1^, remaining constant for higher temperatures. Interestingly, undoped and W-doped VO_2_ pellets have practically the same thermal effusivity in the metallic phase (high temperature). In contrast, in the insulating phase, *ε* of the doped material is higher than the value of the undoped one (see Table 2). This could be expected, since according to previous investigations^27,41^, the transport properties of VO_2_ materials are less (more) affected by W doping in its metallic (insulating) phase, where the entire (local) crystal structure is rutile. It is important to note that the hysteresis width of thermal effusivity decreases in around 1.2 °C with the W doping. This reduction is lower than the corresponding one found in $\Delta H$ for the thermal diffusivity (~3.4 °C).

**Figure 12:**
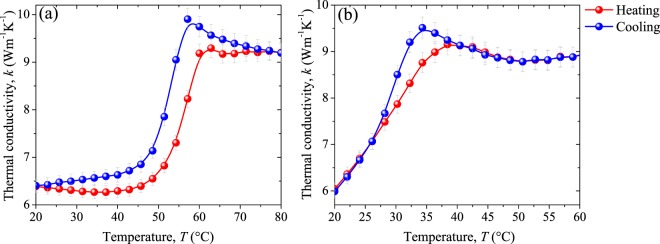
Thermal conductivity of (**a**) undoped VO_2_ and (**b**) W-doped VO_2_ pellets along their MIT. In red (blue) the heating (cooling) process. In gray lines the absolute error.

Based on the results shown in Fig. 11 for thermal diffusivity and thermal effusivity, we calculated the dependence of the thermal conductivity *k* of our VO_2_ pellets with the temperature in the range from 20 °C to 80 °C (see Fig. 11). For the undoped material (Fig. 12a), thermal conductivity follows a similar temperature trend as the thermal effusivity (Fig. 11c). This is related to the small variations obtained in its thermal diffusivity (Fig. 11a). In contrast, for the doped sample, the behavior of both thermal effusivity and thermal diffusivity significantly contribute to the behavior of the thermal conductivity. A remarkable variation in thermal conductivity from 6.33 (7.06) Wm^−1^K^−1^, at room temperature/insulating phase, to 9.19 (8.83) Wm^−1^K^−1^ in the metallic state, was found for undoped (doped) material, which represents a 1.46 (1.25) enhancement of thermal conductivity across the MIT. These results for undoped VO_2_ sample are in good agreement with the theoretical predictions^6,7^ and experimental data^8^ reported previously for our team about VO_2_ thin films. Note also that $\varDelta k={k}_{{\rm{metallic}}}-{k}_{{\rm{insulator}}}$ obtained from the values at the extreme states, exhibits a slight diminution with the W doping, such that ${k}_{{\rm{metallic}}}$(${k}_{{\rm{insulator}}}$) of the undoped material differs in just 4% (11.5%) comparing with the doped one. Note that the behavior of the thermal conductivity through the MIT, particularly in the insulating phase, is clearly affected by the tungsten dopant. This significant feature enables the control of *k* in VO_2_ pellets at low temperatures, such that it could be constant or grow linearly, which can be a relevant feature in specific applications. In addition, we found narrow hysteresis loops for undoped and doped materials, whose average width are of 4.8 °C and 2.4 °C, at transition temperatures around 55.7 °C and 40.5 °C, respectively. It is thus clear that W-doped VO_2_ yields narrower hysteresis loops at lower critical temperatures than pure VO_2_. Notwithstanding, the reduction in grain-coupled size in W-doped VO_2_ displayed in Fig. 3(b) was expected to widen the hysteresis width^58,59^. Several works have demonstrated that in addition to the crystalline grain size of VO_2_, defects produced by the substitutional doping also plays an important role in the hysteresis width^26,59^. Moreover, it has been reported that at low tungsten concentrations (<2.0 at.% W), the narrowing of hysteresis width mainly results from the defects produced by the W dopant. These latest reported results support the significant reduction in the hysteresis width found for *k* of W-doped material at our doping level (0.8 at.% W), and certainly, for the other measured thermal parameters (α, *ε* and *c*_p_), that are presented in Table 3.

**Table 3:** Thermal properties of undoped VO_2_ pellet and W-0.8 at.% VO_2_ pellet. The first column describes the thermal properties under review, the second one shows the MIT characteristics evaluated for each property, such as the variation between the insulating and metallic states (Δ*X*), the critical temperature *T*_*c*_ and hysteresis width Δ*H*.

		Undoped VO_2_	W-doped VO_2_	Undoped - W-doped
Thermal diffusivity *α*	Δα (mm^2^ s^−1^)	0.003 ± 0.003	0.120 ± 0.008	−0.117 ± 0.008
	*T*_*c*_ (°C) ± 0.1	57.1	39.5	17.6
	Δ*H* (°C) ± 0.1	9.5	6.1	3.4
Thermal effusivity *ε*	Δ*ε* (kWs^1/2^ m^−1^ K^−1^)	3.4 ± 0.2	1.8 ± 0.3	1.6 ± 0.4
	*T*_*c*_ (°C) ±0.1	57.8	40.7	17.1
	Δ*H*(°C) ±0.1	4.8	3.6	1.2
Thermal conductivity *k*	Δ*k*(Wm^−1^ K^−1^)	2.9 ± 0.2	2.2 ± 0.3	0.7 ± 0.4
	*T*_*c*_ (°C) ±0.1	55.7	40.5	15.2
	Δ*H* (°C) ±0.1	4.8	2.4	2.4
Specific heat capacity *c*_p_	Δ*c*_p_ (Jg^−1^ K^−1^)	0.85 ± 0.07	0.21 ± 0.08	0.64 ± 0.11
	*T*_*c*_ (°C) ±0.1	55.1	39.5	15.6
	Δ*H*(°C) ±0.1	4.7	4.3	0.4

In columns 3 and 4, are the corresponding values of these latest parameters found for undoped and W-doped VO_2_ samples, and the last column is the difference between columns 3 and 4.

On the other hand, note the anomalous behavior in the trend of the thermal conductivity for both samples, in the form of a peak close to the zone in which the metallic phase is dominant, which is a consequence of the thermal effusivity behavior (Fig. 12c,d) previously observed in the thermal^8^ and optical^13,16^ properties of VO_2_ thin films, being associated to the anomalous energy absorption of VO_2_.

**Figure 13:**
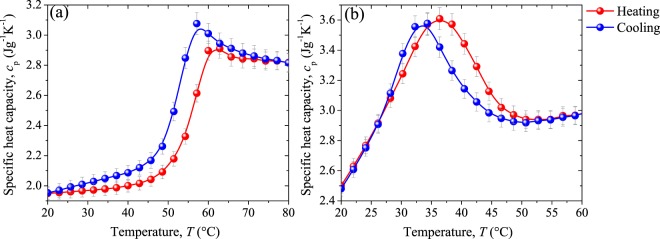
Specific heat capacity of (**a**) undoped VO_2_ and (**b**) W-doped VO_2_ pellets along their heating and cooling processes. In gray lines the absolute error.

Finally, the specific heat capacity *c*_p_ at constant pressure was obtained from the experimental results obtained for the thermal diffusivity and thermal effusivity and plotted in Fig. 13 along the MIT. For both samples, *c*_p_ exhibits a characteristic peak within the MIT during the heating and cooling cycles, appearing at lower temperatures for cooling than for the heating processes. This is consistent with the specific heat capacity of our VO_2_ powders measured by DSC (see Fig. 4) and with previous observations and predictions reported in others works^6,9,10^. Outside the MIT transition, the specific heat capacity of undoped VO_2_ sample keeps nearly constant in agreement with that observed for pure VO_2_ thin films^11,12^, while W-doped VO_2_ pellet show an increasing tendency in the dielectric phase in the studied range of temperature.

Notice also that the change in the specific heat capacity of both samples is totally reversible, showing significant variations between the pure insulating and metal states (44.4 and 20.3%), such that, *c*_p_ in the metallic phase (2.86 and 2.93 Jg^−1^K^−1^) is higher than in the insulating one (1.97 and 2.48 Jg^−1^K^−1^). This behavior can be mainly attributed to the free carrier electronic contribution to the specific heat capacity than to the lattice participation as has been reported in ref.^11^. This indicates that less thermal energy is required to induce the phase transition in W-doped VO_2_ than in undoped VO_2_. However, according to the results presented in Fig. 4, a higher variation in *c*_p_ for the undoped sample could have been expected (see Fig. 13a). This difference can be explained by taking into account that the samples measured by DSC were powders, in contrast to the samples measured by PTR which were pellets, since the energy required to increase the temperature of a given mass of VO_2_, strongly depends on the interconnection between the crystals. Therefore, less energy is required to increase the temperature of VO_2_ pellets than the needed to increase the same mass of VO_2_ in powder form. Note that compared with the other thermal properties, the hysteresis loop width of *c*_p_ presents the lowest variation (0.4 °C) between VO_2_ pure and VO_2_ W-doped.

In Table 3 we summarize the main MIT characteristics of the thermophysical properties under review for undoped VO_2_ and W-0.8 at.% VO_2_ pellets. Note that thermal diffusivity is the only property in which the insulator-metal transition leads to higher variations for the W doped sample than for the undoped one. Moreover, this property is the one which shows the smallest changes through the MIT, accompanied by the widest hysteresis width. Therefore, it is clear that thermal diffusivity of our pure VO_2_ pellet are not strongly affected by the phase transition, but it considerably changes with the tungsten doping. On the other hand, we found a considerable thermal conductivity change between the extreme states for both samples; that is lightly reduced by the 0.8 at.% of tungsten content. Then, one might say that heat conduction switching efficiency tends to decrease with the W doping. However, doping promotes the enhance of the heat propagation in the insulating phase and allows to modify the path that they follow during the transition.

It should be pointed out that the variation of *T*_*c*_ among the W-doped and undoped VO_2_ samples taken from the thermal diffusivity/effusivity differs around 2 °C from those Δ*T*_*c*_ of thermal conductivity/specific heat capacity. Finally, notice that specific heat capacity change decreases with W doping, while its hysteresis width are almost equal for doped and undoped samples.

## Conclusions

We have experimentally measured the hysteretic thermal properties of VO_2_ nanocrystalline compacted powders, by using photothermal radiometry. It has been observed that the 0.8 at.%-W doping decreases the transition temperature up to 15°C and reduces the hysteresis loop width. While thermal diffusivity increases once the metallic phase becomes dominant in the VO_2_ insulating matrix, its other thermal properties tend to decrease with the W doping level, which reduces the contrast of the VO_2_ thermal properties across the phase transition. However, it has been found that at 0.8 at.%-W doping level, the thermal conductivity and thermal effusivity respectively rises in around 11% and 25% in the insulating phase, with respect to their corresponding values for the undoped sample. Furthermore, the characteristic peak of the VO_2_ specific heat capacity has been observed in both the heating and cooling cycles for both samples, such that the doped one requires about 24% less thermal energy than the undoped one to carry out its phase transition. The W doping at 0.8 at.% thus strengths the VO_2_ ability to generate and propagate heat, though with less thermal switching efficiency than pure VO_2_ nanocrystals.

## Supplementary information

Supplementary information
