# Supplementary material for: Measurement of the hysteretic thermal properties of W-doped and undoped nanocrystalline powders of VO2
Source: Sci Rep. 2019 Oct 11;9:14687. doi: 10.1038/s41598-019-51162-4 (PMC6789116; doi:10.1038/s41598-019-51162-4)
Supplement: Supplementary file 1 — Supplementary information [file 41598_2019_51162_MOESM1_ESM.docx]

**Measurement of the hysteretic thermal properties of W-doped and undoped nanocrystalline powders of VO_2_**

C. L. Gomez-Heredia^1^, J. A. Ramirez-Rincon^1^, D. Bhardwaj^2^, P. Rajasekar^2^, I. J. Tadeo^2^, J. L. Cervantes-Lopez^1^, J. Ordonez-Miranda^3*^, O. Ares^1^, A. M. Umarji^2^, J. Drevillon^3^, K. Joulain^3^, Y. Ezzahri^3^, and J. J. Alvarado-Gil^1^

^1^ Departamento de Física Aplicada, Cinvestav-Unidad Mérida, Carretera Antigua a Progreso km. 6, 97310, Mérida, Yucatán, México.

^2^ Materials Research Centre, Indian Institute of Science, 560012, Bengaluru, India.

^3^ Institut Pprime, CNRS, Université de Poitiers, ISAE-ENSMA, F-86962 Futuroscope Chasseneuil, France.

*Corresponding author: jose.ordonez@cnrs.pprime.fr

**Quantification of W atomic percentage**

The XPS spectrum shown in Fig. 2(a) of the manuscript, reveals the presence of three elements: V, W and O. The XPS analysis was conducted considering three different zones of our W-doped VO_2_ pellet. Furthermore, high-resolution profiles of the individual elements have been collected to quantify their amount in the sample (Figs. 2(b) and (c) in the manuscript). Quantification of W is carried out considering the area under the peak and relative sensitivity factors (photoionization cross-section PIC) of the elements summarized in table 1S, as follows:

$$W4f total area=1607.4+1422.0=3029.4$$

$$V2p total area=20232.8+62743.2+10720.4+31565.9=125262.3$$

$$O1s total area=84739.1+55107.5+19101.1=158947$$

Atomic percentage (at.%) of a compound can be obtained by:

| $at.\% of element =\frac{Area of the element/PIC}{\sum Area/PIC}$ | (1S) |
| --- | --- |
| Using equation 1S: |  |

$$at\% of W=\frac{3029.4/0.1449}{\frac{3029.4}{0.1449}+\frac{125262.3}{0.1308}+\frac{158947.7}{0.04}}$$

$$at\% of W=\frac{20906.8233}{4952262.162}=0.0042=0.42\%$$

Tungsten concentration in the sample is thus calculated to be 0.4 at.%. This concentration might be observed because of low penetration of X-rays making XPS as a surface sensitive technique and from some assumptions taken during fitting process: a). The data for O1s, V2p and W4f is assumed to be collected at the same scan rate, b). Data was fitted using O1s peak at 530.0 eV as reference since no data for C1s (adventitious carbon-normally taken as a reference) was available, c). V2p_3/2_ and V2P_1/2_ were fitted at fixed FWHM with an area ratio of 2:1, and also W4f_7/2_ and W4f_5/2_ were fitted at fixed FWHM with an area ratio of 4:3.

Table 1S. Peak parameters taken from the high-resolution profiles for V, O and W of the W-doped sample.

| Peak (eV) | FHWM (eV) | Area | Designation | Photoionization cross-section (PIC) | |
| --- | --- | --- | --- | --- | --- |
| Taken from XPS of V and O (Fig. 2b) | | | | | |
| 515.8 | 1.4 | 20232.8 | V^+3^2p_3/2_ | V2p | 0.1308 |
| 517.2 | 1.4 | 62743.2 | V^+4^2p_3/2_ |  |  |
| 523.2 | 2.1 | 10720.4 | V^+3^2p_1/2_ |  |  |
| 524.6 | 2.1 | 31565.9 | V^+4^2p_1/2_ |  |  |
| 530.0 | 1.4 | 84739.1 | O 1s | O 1s | 0.0400 |
| 531.7 | 1.5 | 55107.5 | O 1s |  |  |
| 533.1 | 1.8 | 19101.1 | O 1s |  |  |
|  | | | | | |
| Taken from XPS of W (Fig. 2c) | | | | | |
| 35.1 | 1.4 | 1607.4 | W^+6^4f_7/2_ | W4f | 0.1449 |
| 37.3 | 1.4 | 1422.0 | W^+6^4f_5/2_ |  |  |
| 40.2 | 2.4 | 5336.4 | W^+6^5pf_5/2_ |  |  |
| 42.1 | 2.4 | 12708.6 | V3p_3/2_ |  |  |
